# Respiratory conditions and health symptoms associated with air pollution amongst children aged six years and below in Melusi Informal Settlement, Tshwane Metropolitan Municipality, South Africa: a cross-sectional study

**DOI:** 10.1186/s12889-024-19324-w

**Published:** 2024-07-30

**Authors:** Moipoti Ruth Makgalemane, Sean Patrick, Joyce Shirinde

**Affiliations:** School of Health Systems and Public Health, Faculty of Health Sciences, University of Pretoria, 31 Bophelo Road, Gezina, 0001 Pretoria South Africa

**Keywords:** Air pollutants, Air pollution, Asthma, Early childhood development centres’, Respiratory conditions, Wheeze

## Abstract

**Background:**

Respiratory conditions and health symptoms associated with air pollution in children are a major public health concern, as their immune systems and lungs are not yet fully developed. This study aimed to assess self-reported respiratory conditions and health symptoms associated with air pollution sources amongst children aged six years and below in Melusi informal settlement, Tshwane Metropolitan Municipality, South Africa.

**Methods:**

With a quantitative cross-sectional study design, parents/caregivers of children aged six years and below (*n = 300*) from eight Early Childhood Development Centres were invited to participate in the study. This study employed complete sampling, and data was collected using the modified International Study of Asthma and Allergies in Children. The chi-square and multiple logistic regression models were used to analyze data, with *p* < 0.05 in the adjusted odds ratios considered as being statistically significant.

**Results:**

Three models were run to examine the predictors of wheezing in the past 12 months, dry cough, and itchy-watery eyes. The model for asthma was excluded, as only seven participants reported having asthma. Wheeze in the past 12 months was associated with participants living in the area for more than three years (OR 2.96 95%CI: 1.011–8.674). Furthermore, having a dog in the house in the past 12 months was associated with wheeze in the past 12 months (OR 5.98 95%CI: 2.107–16.967). There was an association between duration of stay in a residence and dry cough prevalence (OR 5.63 95%CI: 2.175–14.584). Trucks always or frequently passing near homes was associated with itchy-watery eyes (OR 3.27 95%CI: 1.358–7.889). 59% (59%) of participants perceived the indoor air quality in their homes to be good, while 6% perceived it as poor. In contrast, 36% of participants perceived the outdoor air quality to be good, and 19.7% perceived it as poor.

**Conclusion:**

The association between perceived air pollution exposure, self-reported respiratory conditions, and health symptoms amongst children is complex. Further research is required to better understand the multifaceted nature of air pollution and its impact on the health of children.

**Supplementary Information:**

The online version contains supplementary material available at 10.1186/s12889-024-19324-w.

## Background

The adverse health effects of air pollution remain a public health concern globally [1]. Exposure to both indoor and ambient (outdoor) air pollution has been linked to the causation of numerous respiratory conditions such as asthma, chronic obstructive pulmonary diseases (COPDs), bronchiolitis and lung cancer [2, 3]. Cardiovascular diseases, cutaneous diseases and nervous system dysfunctions have been found to be associated with long term exposure to air pollution [4–6].

Air pollutants are known to contribute immensely to adverse health effects associated with air pollution [7]. These air pollutants are associated with a range of activities and sources such as, traffic related emissions, burning of fossil fuels, industrial activities, and agricultural activities [7]. Exposure to air pollutants such as Particulate Matter (PM), Ozone (O_3_), Nitrogen (NO_2_), Sulphur Dioxide (SO_2_) and Traffic Related Air Pollution (TRAP) have been implicated in the myriad disease process [8] and are currently ranked as the fifth highest risk factor for mortality worldwide [9].

Exposure to air pollution may lead to a range of inflammatory changes in the airways, which vary depending on the type of air pollutants that an individual was exposed to [7]. The severity of respiratory conditions and health symptoms in both the general and vulnerable populations are affected by the type of air pollutant exposure [7]. Research studies indicate that early life exposure to air pollutants is linked to restrictive lung growth and airway obstruction [7].

Children are overly sensitive and most susceptible to the adverse health effects of air pollution, as their lungs, immune systems, and metabolic functions are still rapidly developing [10, 11]. Studies involving general populations of children and adults showed that exposure to particulate pollution was associated with breathlessness, cough, and wheezing [12, 13]. The South California Children’s Health Study identified air pollution in homes and schools as a significant contributor to incident asthma in kindergarten and first-grade students who were asthma and wheeze-free at the start of the study [14]. Children, in comparison to adults, inhale a higher volume of air per body weight [15]. In children, evidence suggests that PM, NO_2_ and O_3_ are associated with health conditions such as airway inflammation, lung function deficits and respiratory conditions such as asthma [11, 16].

Lower respiratory diseases are the most frequent causes of hospital admission in children worldwide, particularly in developing countries. A Portugese study found that environmental context (urban, suburban or rural), gender and family asthma history showed clear associations with asthma prevalence, namely non-rural location, male gender, and being the child of an asthmatic parent were found to be risk factors [17].A Ugandan study found that 41% of children under five who were hospitalized with symptoms of acute respiratory illness actually had asthma or similar bronchospastic conditions [18]. In Ethiopia, a study was conducted on respiratory symptoms and associated risk factors among children under five. The study found that the prevalence of respiratory symptoms were 37.5% at [95% (CI: 34.3–41)] [19].

A study among schoolchildren in Durban was conducted to assess the correlation between ambient air pollutants and respiratory outcomes among Schoolchildren [20]. The study participants were selected from the highly industrialized South and non-industrial North regions. Results demonstrated that air pollutants Sulphur Dioxide (SO_2_ ) were higher in the South than they were in the North, and that PM was the same across all regions [20]. Therefore, persistent conditions such as asthma and Airway Hyperreactivity (AHR) were higher among children from schools in the South than it was for those in the North. South Africa has the highest ambient air pollution exposure in the Sub-Saharan Africa (SSA) region, accounting for approximately 14 356 confirmed cases annually, with majority of those cases being children [21]. A study conducted in Nairobi reported that asthma was more prevalent in children attending school at an informal settlement [22].

Environmental variables, particularly in informal settlements, have a major impact on respiratory and allergy diseases in children aged six years and below. In this age range, wheezing is a common respiratory condition that is frequently made worse by exposure to air pollutants from heavy traffic, especially trucks that pass by these places and release large amounts of particulate matter and other pollutants into the air [23]. Children exposed to high amounts of allergens, particularly those from domestic pets like dogs, which add to indoor allergen load, often experience itchy, watery eyes, which are symptomatic of allergic conjunctivitis [24]. Children who live in highly populated informal settlements, where air quality is damaged by both indoor and outdoor pollution sources, are more likely to suffer from dry cough, which is frequently caused by respiratory infections or chronic irritation [25]. The kind of home and its location have a big impact on the quality of the air; kids who live in poorly ventilated homes in busy neighborhoods are more likely to experience these symptoms [26]. Understanding these environmental determinants is crucial for developing targeted public health interventions to mitigate respiratory and allergic diseases in vulnerable populations. The aim of this study was to assess the self-reported respiratory conditions and health symptoms associated with perceived air pollution amongst children aged six years and below through a questionnaire completed by their parents/caregivers, in Melusi informal settlement in Tshwane Metropolitan Municipality, South Africa.

## Methods

### Study setting

**Fig. 1:**
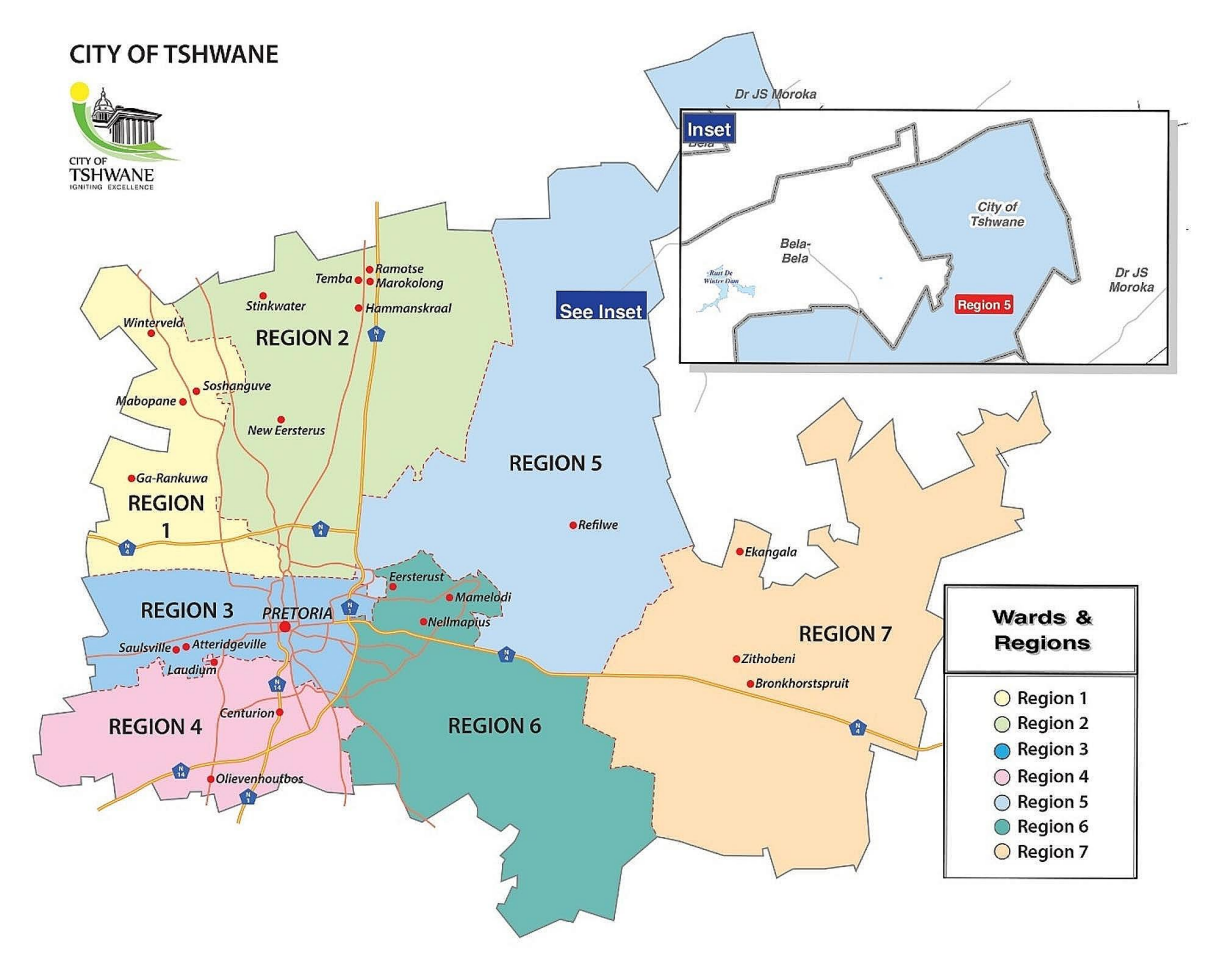
Map illustrating the City of Tshwane metropolitan municipality

The study was conducted in Melusi informal settlement which is located in the West of Pretoria within the City of Tshwane Metropolitan Municipality, Region 3, see Fig. 1 below. The City of Tshwane is home to approximately 4.0 million citizens, as of the 2022 Census Data. 3.3 million of the 4.0 million citizens of the City of Tshwane are predominantly black Africans [27]. Melusi informal settlement forms part of the 227 informal settlements located in the City of Tshwane Metropolitan Municipality [28]. The municipality forms the local government of the Northern Gauteng Province, South Africa.

### Study design, population and sample selection

A quantitative cross-sectional study was conducted. In 2023, there were eight Early Childhood Development Centres’ in the area. All school principals were approached and asked to participate by allowing us to invite the parents/caregivers of children attending their schools. In addition, all parents/caregivers of eligible children were invited to participate to achieve a comprehensive census. This study used a complete sample strategy, with the study sample being made up of 464 children.

### Health outcomes

In this study, data was collected using a modified International Study of Asthma and Allergy in Children (ISAAC) questionnaire (see Supplementary File 1) to assess self-reported respiratory conditions and health symptoms associated with exposure to air pollution. The following health outcomes were identified on the basis of positive answers that were given from the questionnaire: asthma [“Has your child ever had asthma?”], wheeze in the past 12 months [“Has your child had wheezing or whistling in the chest in the past 12 months?”], dry cough [“In the past 12 months, has your child had a dry cough at night, apart from a cough associated with a cold or chest infection?”], itchy-watery eyes [“In the past 12 months, has this nose problem been accompanied by itchy-watery eyes?”].

### Air pollution sources

Air pollution sources included: mode of transport for children (walk, taxi/bus, motor car, combination and other). The type of fuel used for cooking and heating (electricity, gas, paraffin, open fires and other-please specify) were also asked. ETS exposure at home, school, in the car, and at the restaurant in the past 30 days (never/1–6 days/7–10 days/16–20 days and more than 20 days) were questioned. Smoking father/male guardian, mother/female guardian (yes/no) and any other individual residing in the residence apart from the parents/caregivers were identified as air pollution sources. Frequency of trucks passing through neighborhoods (never/seldom/frequently through the day/almost all day) was included as an air pollution source.

### Confounders

Probable confounding variables included: sex(male/female), suburb/township/section where the child lives, period lived in residential area (< 6 months/6 to 12 months/1 to 2 years or 3 years or longer). Duration to the nearest clinic or hospital from your home (15 minutes’ walk or 5-minute drive/1 hour walk or 15-minute drive/ more than an hour’s walk or more than 30-minute drive), type of house your child lives in (brick/mud/corrugated iron/combination/other), number of rooms in the household, running water in the house (yes/no), type of regular food consumption from 15 food items e.g., meat, fruits, vegetables, nuts (never or occasionally/once or twice per week/three or more times per week). Child’s absenteeism from school (never or occasionally/once or twice per week/three or more times a week) duration of child watching television (< 1 h/1 hour but less than 3 h/3hours but less than 5 h/5 hours or more), paracetamol usage in the past 12 months (never/at least once a year/at least once per month), younger and older siblings, pets (dog and/or cat) in or around the household (yes/no) were also identified as probable confounding variables.

### Perceptions of air pollution and air quality

Perceptions of air pollution and air quality were defined as those who answered to all eight questions:

1. “How would you rate the indoor air quality in your home?”. For this question, the parents/caregivers could select one of the following three options: (a) good, (b) average or (c) poor. Included in the definition of indoor air quality were those who indicated good or poor indoor air quality.
2. “How would you rate the outdoor air quality in your home?”. For this question, the parents/caregivers could select one of the following three options: (a) good, (b) average or (c) poor. Included in the definition of outdoor air quality were those who indicated good or poor indoor air quality.
3. “Improving the environment is the responsibility of every citizen?”. For this question, the parents/caregivers could select one of the following five options: (a) strongly agree, (b) agree, (c) undecided, (d) disagree or (e) strongly disagree. Included in the definition of improving the environment were those who indicated strongly agree.
4. “Recycling programs should be put in place and promoted across the whole city?”. For this question, the parents/caregivers could select one of the following five options: (a) strongly agree, (b) agree, (c) undecided, (d) disagree or (e) strongly disagree. Included in the definition of recycling programs were those who indicated strongly agree.
5. “I am actively involved in cleaning up the environment?”. For this question, the parents/caregivers could select one of the following five options: (a) strongly agree, (b) agree, (c) undecided, (d) disagree or (e) strongly disagree. Included in the definition of active involvement were those who indicated strongly agree.
6. “The pollution is out of my control, and I cannot do anything to change it?”. For this question, the parents/caregivers could select one of the following five options: (a) strongly agree, (b) agree, (c) undecided, (d) disagree or (e) strongly disagree. Included in the definition of pollution is out of my control were those who indicated strongly agree.
7. “If I knew how to better contribute to a cleaner environment, I would take action?”. For this question, the parents/caregivers could select one of the following five options: (a) strongly agree, (b) agree, (c) undecided, (d) disagree or (e) strongly disagree. Included in the definition of contribute to a cleaner environment were those who indicated strongly agree.
8. “I do not see the pollution as a health problem?”. For this question, the parents/caregivers could select one of the following five options: (a) strongly agree, (b) agree, (c) undecided, (d) disagree or (e) strongly disagree. Included in the definition of pollution as a health problem were those who indicated strongly disagree.

### Statistical analysis and data management

Data was captured using Microsoft Excel. Stata Statistical Software: Release 18. College Station, TX: StataCorp LLC. was utilized for data analysis. Simple descriptive statistics and frequencies were used to calculate percentages for demographic characteristics, and air pollution sources. Observations that were not marked or were left blank were set as missing, which resulted in each question having a slightly different sample size. Univariate and multiple logistic regression (LRA) were applied, and missing values were automatically excluded from LRA. Crude and adjusted odds ratios (OR) and 95% Confidence Intervals (CIs) were calculated to assess the likelihood of self-reported respiratory conditions (asthma), health symptoms (wheeze in the past 12 months, dry cough, and itchy-watery eyes) and air pollution and air quality perceptions. The respiratory condition (asthma), health symptoms (wheeze in the past 12 months, dry cough, and itchy-watery eyes), air quality perceptions and confounding variables that had p-values estimated at less than or equal to 0.25 in the univariate analysis were included in the LRA analysis. In the LRA analysis, p-values being estimated at less than 0.05 were considered as being statistically significant.

### Ethics

#### Ethical approval

was obtained from the Research Ethics Committee of the Faculty of Health Sciences, University of Pretoria (Ethics Numbers: 363/2022 and 382/2023). Permission to conduct the study was granted by the Gauteng Department of Basic Education (GDoBE). Invitation letters with information pertaining to the study were firstly given to the principals of the ECDs, to gain permission and access for conducting the study on their premises as well as interacting with the parents/caregivers of the children. Once permission was granted from the ECDs principals, informed consent forms were then given to the parents/caregivers of the children prior data collection. Parents/caregivers who were unable to read or write had witnesses, either the staff at the ECDs or individuals residing in the parents/caregivers’ households, to fill in the informed consent and questionnaire, and sign on their behalf. Participation in the study was entirely voluntary, and informed consent was given to the parents/caregivers’ prior data collection, with clear instructions that the study was voluntary and not mandatory, and that they could leave the study at any time. Participants were ensured confidentiality, privacy, anonymity, and non-maleficence.

## Results

### Demographic characteristics, health outcomes and air pollution sources

Of the *n* = 464 children, the parents/caregivers of *n* = 300 children completed the modified ISAAC questionnaire at the ECDs (65% response rate). The study focused only on the children that returned the signed informed consent forms and questionnaires. The remaining *n* = 164 children did not participate in the study. The ECDs principals and teachers stated that the majority of the kids had left the ECDs to go to other areas of residence and ECDs during the period of data collection. The *n* = 300 completed questionnaires were used in the data analysis, inclusive of those that had missing data due to the low response rate.

The demographic characteristics of the children’s health outcomes and air pollution sources are summarized in Table 1. The prevalence of having had wheeze ever in the past and sneezing or runny nose was 6% and 20% respectively. Males accounted for (45%) of the participants, and females (50%). Majority of the children lived in the Gomora area (29%). Half of the children had mentioned that they have lived in their specified residential area for a minimum of 3 years or more (50%). 17% (17%) of the participants had a father or male guardian that smoked and only 2% of mothers or female guardians of the participants were also smokers. In addition, for Environmental Tobacco Smoke (ETS) exposure in the home, car/transport, and restaurant, 18% of the participants were exposed to ETS in the home, 4% in the car/transport and 4% in the restaurant. 26% (26%) of the participants most frequently used electricity for cooking, 52% most frequently used gas, 18% most frequently used paraffin, 2% most frequently used open fires and 1% most frequently used other means of cooking in their households. For heating, 25% most frequently used electricity, 20% most frequently used gas, 7% most frequently used paraffin, 35% most frequently used open fires and 4% used other means of heating. Trucks passing by in the residential area were reported as 34% never, 11% seldom, 29% frequently throughout the day and 18% almost all-day Table 1.

**Table 1:** Sociodemographic, clinical characteristics and air pollution sources (*n = 300*)

Variables	Total	%
**Sex**		
Male	134	44.7
Female	150	50.0
Missing	16	5.3
**Residential Area**		
Melusi	47	15.7
Gomora	87	29.0
Hercules	40	13.0
Booysens Ext 4	29	9.7
Missing	97	32.3
**Period lived in residential area**		
< 6 months	28	9.3
6–12 months	31	10.3
1–2 years	77	25.7
± 3 years	149	49.7
Missing	15	5.0
**Wheeze ever**		
Yes	19	6.3
No	245	81.7
Missing	36	12.0
**Sneezing or Runny Nose**		
Yes	61	20.3
No	17	5.7
Missing	222	78.0
**Itchy-Watery Eyes**		
Yes	20	6.7
No	42	14.0
Missing	238	79.3
**Smoking father or male guardian**		
Yes	52	17.3
No	239	79.7
Missing	9	3.0
**Smoking mother or female guardian**		
Yes	5	1.7
No	287	95.7
Missing	8	2.7
**ETS exposure of children at home**		
Yes	55	18.3
No	193	64.3
Missing	52	17.3
**ETS exposure of children at school**		
Yes	1	0.3
No	171	57.0
Missing	128	42.7
**ETS exposure of children in car/transport**		
Yes	13	4.3
No	167	55.7
Missing	120	40.0
**ETS exposure of children in restaurant**		
Yes	11	3.7
No	159	53.0
Missing	130	43.3
**Mode of transport**		
Walk	259	86.3
Taxi/Bus	8	2.7
Motor car	17	5.7
Combination	6	2.0
Other	1	0.3
Missing	9	3.0
**Fuel used for cooking**		
Electricity	79	26.3
Gas	155	51.7
Paraffin	55	18.3
Open fires	5	1.7
Others	2	0.7
Missing	4	1.3
**Fuel used for heating**		
Electricity	76	25.3
Gas	60	20.0
Paraffin	21	7.0
Open fires	104	34.7
Others	11	3.7
Missing	28	9.3
**Trucks passing near home on weekdays**		
Never	102	34.0
Seldom	33	11.0
Frequently through the day	88	29.3
Almost all day	54	18.0
Missing	23	7.7

Tables 2, 3 and 4 summarize the results of Multiple Logistic Regression models that were run to examine the predictions of wheezing in the past 12 months, dry-cough, and itchy-watery eyes. The asthma model was excluded because there were only seven participants that had asthma, and no significant associations with any factors in this study.

**Table 2:** Prevalence of wheeze in the past 12 months amongst children and its predictors

Variables	*N*(%)^a^	Crude OR (95% CI)^b^	*p*-value	Adjusted OR (95% CI)^b, c^	*p*-value
**Residence**					
Lived in the area for < 3 years	150(57.0)	1		1	
Lived in the area for > 3 years	35(13.3)	1.87 (0.753–4.620)	**0.178**	**2.96 (1.011–8.674)**	**0.048**
**Distance to nearest clinic/hospital**					
1 h or less	189(71.9)	1			
More than 1 h walking	67(25.5)	1.24 (0.636–2.433)	0.524	1.37 (0.595–3.178)	0.456
**House**					
Other materials	72(28.7)	1		1	
Corrugated Iron or Brick	179(71.3)	0.78 (0.402–1.513)	0.465	0.6 (0.270–1.348)	0.218
**Fuel used for heating**					
Other fuels	32(11.9)	1		1	
Electricity	68(25.9)	1.48 (0.444–4.898)	0.525	1.87 (0.345–10.084)	0.469
No electricity	192(73.0)	1.36 (0.443–4.158)	0.591	1.48 (0.303–7.245)	0.627
**Trucks passing by**					
Seldom or never	122(50.4)	1		1	
Frequently or always	120(49.6)	0.87 (0.477–1.598)	0.659	1.34 (0.634–2.823)	0.445
**Cat in the home in the past 12 months**					
No	248(96.5)	1		1	
Yes	9(3.5)	1.53 (0.400-5.867)	0.532	0.36 (0.057–2.317)	0.284
**Dog in the home in the past 12 months**					
No	244(94.6)	1		1	
Yes	21(8.1)	3.73 (1.670–8.344)	**0.001**	**5.98 (2.107–16.967)**	**0.001**
**Smoking Father**					
No	211(82.1)	1		1	
Yes	46(17.9)	0.82 (0.362–1.877)	0.646	0.55 (0.190–1.615)	0.28

^**a**^ An Analysis was conducted using complete case analysis, proportions for categories do not add up to a 100% due to missing values and non-bivariate response

^**b**^ Values that are statistically significant at less than 0.25 for the crude OR and less than 0.05 for the adjusted OR are bolded

^**c**^ Model adjusted for all variables in this table

**Table 3:** Prevalence of dry cough among children and its predictors

Variables	*N*(%)^a^	Crude OR (95% CI)^b^		*p*-value	Adjusted OR (95% CI)^b, c^		*p*-value
**Residence**							
Lived in the area for < 3 years	157(56.5)	1			1		
Lived in the area for > 3 years	34(12.2)	4.05 (1.923–8.548)		**0.000**	**5.63 (2.175–14.584)**		**0.000**
**Distance to nearest clinic/hospital**							
1 h or less	200(71.9)	1			1		
More than 1 h walking	70(25.2)	1.63 (0.649–2.782)		0.077	1.57 (0.795–3.099)		0.194
**House**							
Other materials	76(28.4)	1			1		
Corrugated Iron or Brick	192(71.6)	1.10 (0.635–1.910)		0.731	1.04 (0.524–2.047)		0.919
**Trucks passing by**							
Seldom or never	133(51.4)	1			1		
Always or frequently	126(48.6)	0.82 (0.501–1.359)		0.449	0.72 (0.385–1.342)		0.300
**Cat in the home in the past 12 months**							
No	261(96.0)	1			1		
Yes	11(4.0)	0.61 (0.161–2.303)		0.465	0.22 (0.034–1.375)		0.105
**Dog in the home in the past 12 months**							
No	246(89.1)	1			1		
Yes	30(10.9)	1.14 (0.519–2.496)		0.746	1.64 (0.615–4.349)		0.324
**Smoking Father**							
No	226(82.5)						
Yes	48(17.5)	1.05	0.556–1.966	0.890	1.32	0.578–3.002	0.513

^**a**^ An Analysis was conducted using complete case analysis, proportions for categories do not add up to a 100% due to missing values and non-bivariate response

^**b**^ Values that are statistically significant at less than 0.25 for the crude OR and less than 0.05 for the adjusted OR are bolded

^**c**^ Model adjusted for all variables in this table

**Table 4:** Prevalence of itchy-watery eyes among children and its predictors

Variables	*N*(%)	Crude OR (95% CI)^a^	*p*-value	Adjusted OR (95% CI)^b, c^	*p*-value
**Residence**					
Lived in the area for < 3 years	158(55.6)	1		1	
Lived in the area for > 3 years	36(12.7)	13.75 (5.767–32.801)	**0.000**	**38.72 (10.336-145.043)**	**0.000**
**Distance to nearest clinic/hospital**					
1 h or less	206(72.5)	1		1	
More than 1 h walking	7225.4)	2.23 (1.257–3.947)	0.006	2.41 (0.991–5.873)	0.052
**House**					
Other materials	76(27.9)	1		1	
Corrugated Iron or Brick	196(72.1)	0.73 (0.408–1.296)	0.280	0.58 (0.248–1.377)	0.219
**Trucks passing by**					
Seldom or never	134(51.0)	1		1	
Always or frequently	129(49.0)	1.67 (0.970–2.878)	**0.064**	**3.27 (1.358–7.889)**	**0.008**
**Dog in home in the past 12 months**					
No	250(89.3)	1		1	
Yes	30(10.7)	0.55 (0.204–1.504)	0.247	0.86 (0.195–3.777)	0.840
**Smoking Father**					
No	226(81.9)	1		1	
Yes	50(18.1)	0.56 (0.257–1.207)	0.138	0.70 (0.224–2.212)	0.547

^**a**^ An Analysis was conducted using complete case analysis, proportions for categories do not add up to a 100% due to missing values and non-bivariate response

^**b**^ Values that are statistically significant at less than 0.25 for the crude OR and less than 0.05 for the adjusted OR are bolded

^**c**^ Model adjusted for all variables in this table

### Prevalence of wheeze in the past 12 months and air pollution among participants

Living in the residential area for more than three years was associated with wheeze (OR 2.96 95%CI: 1.011–8.674). Having a dog in the home was associated with wheeze in the past 12 months (OR 5.98 95%CI: 2.107–16.967). (Table 2).

### Prevalence of dry cough and air pollution among participants

Table 3 indicates that there were associations between the duration of stay in a residence and dry cough prevalence. The results suggest that individuals who lived in the area for more than three years have 5.63 times higher odds of having a dry cough compared to those whose residence is not specified (95%CI: 2.175–14.584). (Table 3).

### Prevalence of itchy-watery eyes and air pollution among participants

Table 4 indicates that there is a strong positive association between duration of stay in a residence, and prevalence of itchy-watery eyes. That is, participants who lived in the area for more than three years have much higher odds of experiencing itchy-watery eyes (OR 38.72 95%CI: 10.336-145.043). There is a positive association between trucks passing by frequently or always and the itchy-watery eyes. Participants who lived near trucks passing by frequently or always had 3.27 times odds (95%CI: 1.358–7.889) of having itchy-watery eyes (Table 4).

### Perceptions of air pollution and its association with self-reported respiratory conditions and health symptoms

Air pollution and air quality perceptions were assessed by asking participants to rate the air quality in their homes, communities, and key action areas to tackle the issue of air pollution. 59% 59% of participants stated that the air quality in their homes was good, 31.7% stated it was average, 6% stated that it was poor, and 3.3% stated nothing at all. With outdoor/community air quality, 36% participants mentioned that the outdoor air quality was good, 39.7% stated it was average, 19.7% stated that it was poor, and 4.7% did not state anything at all.

Participants were also asked for their opinions on improving the environment, recycling, personal activities to clean the environment, actions taken to combat air pollution, knowledge of air pollution and its complexities and not seeing air pollution as an issue. “*Improving the environment is the responsibility of every citizen,”* was respectively responded as, 60.7% strongly agreeing, 24.7% agreeing, 2.7% being undecided, 1.7% disagreeing and 9.3% not stating anything at all.

For the recycling programs, participants were asked *“Recycling programs should be put in place and promoted across the whole city.”* Majority of the participants 46.7% strongly agreed, while 29.3% agreed, 5.7% were undecided, 5% disagreed, 1.7% strongly disagreed and 11.7% did not state anything. For personal active involvement in cleaning the environment “*I am actively involved in cleaning up the environment*,” had 27% participants strongly agreeing, 37% agreeing, 15.3% undecided, 6.3% disagreeing, 1% strongly disagree, and 13.3% not stating anything.

For “*The pollution is out of my control, and I cannot do anything to change it,”* statement, 16.7% strongly agree, 16% agree, 9.3% were undecided, 29.3% disagree, 15.7% strongly disagree and 13% not stating anything. With the ‘If I knew how to better contribute to a cleaner environment I would take action,” statement, 43.3% strongly agree, 33% agree, 2% were undecided, 2.3% disagree and 1% strongly disagree, and 18.3% stated nothing.

The *“I do not see the pollution as a health problem*,” statement had 5.7% strongly agreeing, 8.3% agree, 4.3% being undecided, 21.3% disagreeing, 35.7% strongly disagreeing and 24.7% not stating anything.

## Discussion

The study aimed to assess the self-reported respiratory conditions and health symptoms associated with air pollution in an informal settlement in Tshwane Metropolitan Municipality. While there was no compelling evidence for associations between perceived air pollution and reported respiratory conditions and health symptoms, a small-scale effect was possible, due to the living conditions in the informal settlement. This may have been relatively influenced by the small sample size as well as the low response rate. Our study adopted the modified ISAAC questionnaire. Most asthma and wheezing related studies have used the modified ISAAC questionnaire among 6–7 and 13–14 year olds [29]. However, other studies have stated that, the validity of the modified ISAAC questionnaire was not professionally researched [30]. Globally, the prevalence of asthma and wheezing is much higher in younger children than it is in older children [31]. For this present study, asthma was excluded, as it had no associations with any factors in the study, as only seven participants reported to having asthma, even though other studies concluded that asthma and wheezing was much higher in younger children [31].

With this current study, wheezing was associated with residing in a specified residential area for more than three years, (OR 2.96 95%CI: 1.011–8.674). A study conducted by Sheuya^32^ and Webber, Carpiniello, Oruwariye and Appel^33^ reported that high levels of wheezing are mostly observed in underserved communities than in rural, semi-urban and urban settings [32, 33]. Having a dog in the house for the past 12 months was associated with wheeze in children (OR 5.98 95%CI: 2.107–16.967). On the contrary, research done in Japanese households, conducted by Taniguchi et al.^34^ stated that, dog or cat ownership did not increase the risks of wheezing in toddlers [34]. It is however crucial to consider the potential underreporting of symptoms, especially in children, which could be influenced by factors such as parent/caregiver awareness and healthcare-seeking behaviour. A study by Seneviratne and Gunawardena^29^ reported that the issue of parental reporting of wheezing lead to under or overestimation of the wheezing illness in children [29].

Participants who stayed at a residential area for more than three years had 5.63 times higher odds of having a dry cough compared to those whose residence is not specified (95%CI: 2.175–14.584). Whereas another study conducted in Slovakia showed no significance in dry cough parameters between children living in urban or rural areas [35]. In the capital city of India, a study was conducted in Delhi, with findings compared with those of rural West Bengal and Uttaranchal. Children in Delhi accounted for 6.6% of dry cough cases, while the prevalence was the lowest in south Delhi [36]. For this current study, trucks passing near homes always or frequently had an association with itchy-watery eyes. A study conducted by Shirinde, Wichmann and Voyi^37^ also concluded that, children that stayed in areas where trucks passed by frequently had rhinitis ever, current rhinitis and current rhinoconjunctivitis [37

Perceptions of air pollution and air quality were also assessed. Majority of the participants (59%) rated their indoor air quality was good, in comparison to those that rated it poor at 3.3%. In terms of outdoor air quality, 36% participants rated it good, while 19.7% stated that it was poor. Perceptions of air pollution and air quality in terms of improving the environment, recycling activities, personal activities to clean the environment, actions to combat air pollution, knowledge of air pollution and its complexities and not seeing air pollution as an issue were also assessed.

Majority (60.7%) of the participants believed that it was every citizens responsibility to clean up the environment, with a study by Rives, Elshorbany and Kaylor^38^ supporting that statement, revealing that most participants (74%) were concerned about air quality to some degree. With recycling, almost half (46.7%) of the participants stated that recycling programs should be implemented in the whole city [38]. Supporting this, a study conducted in the state of Massachusetts found that recycling led to air quality improvement [39] For the personal involvement of cleaning up the environment, only 37% agreed, and not strongly agreed. Similarly, a study conducted by Sennes et al.^40^ reported that, majority of the participants (58%) stated that their personal actions were consistent with commitment to the local environment  [40 Majority of the participants (29.3%) were undecided about the statement of air pollution being out of their control and that they cannot do anything about it. Xu et al.^41^ found results like this current study, that when people felt powerless about an issue which they had to bear with, they tended to allocate little concern to it  [41] Contributing to a cleaner environment had 43.3% of participants strongly agreeing that, if they knew how to better contribute to a cleaner environment, they would act. Ramirez, Ramondt, Van Bogart and Zuniga^42^^44^ had participants of mothers’ state they searched about air pollution and its consequences, especially because their children were vulnerable [42. Not seeing air pollution as a cause for concern was strongly disagreed upon by majority of the participants (24.7%). Other studies also agreed with this current study results, that air pollution is a cause for concern  [43, 44]

The current study had some limitations. Firstly, this research study deployed a cross-sectional study design, which means data was collected at one point in time. The usage of a cross-sectional study does not allow exploration of the temporal relationship between factors and the outcome [25]. Secondly, the relatively low response rate, while common in studies involving mobile populations in informal settlements, raises concerns about the representativeness of the sample. Non-responses could be due to several factors, including parents’ or caregivers’ availability and willingness to participate, potentially leading to a non-response bias. Lastly, data collected relied on self-reporting through questionnaires, which may introduce recall bias, particularly for clinical characteristics and exposure to air pollution. Parents or caregivers might not accurately recall past health conditions or symptoms in their children, impacting the validity of the findings.

## Conclusion

In conclusion, our study contributes valuable insights into the complex relationship between perceived air pollution exposure and self-reported respiratory health among children in Melusi informal settlement. While some associations were observed, further research is needed to better understand the multifaceted nature of air pollution’s impact on children’s health, especially in LMICs. Our findings underscore the importance of targeted interventions and policies aimed at reducing air pollution and its adverse health effects on children, not only in our region but also in similar settings globally.

### Recommendations

Develop and promote educational programs targeting parents, caregivers, and community members about the health risks associated with air pollution. Emphasize the importance of smoke-free environments, safe cooking practices, and reducing exposure to outdoor pollutants. Develop policies and programs aimed at improving the long-term housing stability of families in informal settlements. Stable housing can reduce exposure to environmental hazards and contribute to better overall living conditions, in line with.

the targets in Sustainable Development Goals (SDGs) 3 and 11.

Support further research in informal settlements to better understand the complex relationship between air pollution and respiratory health among children. Longitudinal studies and comprehensive air quality monitoring are needed to inform evidence-based policies and interventions.

### Electronic supplementary material

Below is the link to the electronic supplementary material.

Supplementary Material 1

## Data Availability

We did not receive ethical approval to share raw data publicly. The data belongs to the University of Pretoria. The raw data for this current study is available from the University of Pretoria on reasonable request and approval by the Research Ethics Committee, of the Faculty of Health Sciences, University of Pretoria.

## Abbreviations

AHR: Airway Hyperreactivity
CI: Confidence Interval
COPDs: Chronic Obstructive Pulmonary Diseases
ECDs: Early Childhood Development Centres
ETS: Environmental Tobacco Smoke
GDoBE: Gauteng Department of Basic Education
ISAAC: International Study of Asthma and Allergies in Childrens
LMICs: Lower-Middle Income Countries
NO_2_: Nitrogen
O_3_: Ozone
OR: Odds Ratio
PM: Particulate Matter
SO_2_: Sulphur Dioxide
SDGs: Sustainable Development Goals
SSA: Sub-Saharan Africa
TRAP: Traffic Related Air Pollution

## References

[1] Jiang XQ, Mei XD, Feng D. Air pollution and chronic airway diseases: what should people know and do? J Thorac Dis. 2016;8(1):E31–40. 10.3978/j.issn.2072-1439.2015.11.50. 26904251 10.3978/j.issn.2072-1439.2015.11.50 PMC4740163

[2] Orellano P, Reynoso J, Quaranta N, Bardach A, Ciapponi A. Short-term exposure to particulate matter (PM10 and PM2.5), nitrogen dioxide (NO2), and ozone (O3) and all-cause and cause-specific mortality: systematic review and meta-analysis. Environ Int. 2020;142:105876. 10.1016/j.envint.2020.105876. 32590284 10.1016/j.envint.2020.105876

[3] Zheng XY, Orellano P, Lin HL, Jiang M, Guan WJ. Short-term exposure to ozone, nitrogen dioxide, and sulphur dioxide and emergency department visits and hospital admissions due to asthma: a systematic review and meta-analysis. Environ Int. 2021;150:106435. 10.1016/j.envint.2021.106435. 33601224 10.1016/j.envint.2021.106435

[4] Huangfu P, Atkinson R. Long-term exposure to NO2 and O3 and all-cause and respiratory mortality: a systematic review and meta-analysis. Environ Int. 2020;144:105998. 10.1016/j.envint.2020.105998. 33032072 10.1016/j.envint.2020.105998 PMC7549128

[5] Manisalidis I, Stavropoulou E, Stavropoulos A, Bezirtzoglou E. Environmental and Health impacts of Air Pollution: a review. Front Public Health. 2020;8:14. 10.3389/fpubh.2020.00014. 32154200 10.3389/fpubh.2020.00014 PMC7044178

[6] Chen J, Hoek G. Long-term exposure to PM and all-cause and cause-specific mortality: a systematic review and meta-analysis. Environ Int. 2020;143:105974. 10.1016/j.envint.2020.105974. 32703584 10.1016/j.envint.2020.105974

[7] Adamkiewicz G, Liddie J, Gaffin JM. The respiratory risks of Ambient/Outdoor Air Pollution. Clin Chest Med. 2020;41(4):809–24. 10.1016/j.ccm.2020.08.013. 33153697 10.1016/j.ccm.2020.08.013 PMC7665094

[8] Schraufnagel DE, Balmes JR, Cowl CT, et al. Air Pollution and Noncommunicable diseases: a review by the Forum of International Respiratory Societies’ Environmental Committee, Part 1: the Damaging effects of Air Pollution. Chest. 2019;155(2):409–16. 10.1016/j.chest.2018.10.042. 30419235 10.1016/j.chest.2018.10.042 PMC6904855

[9] Cohen AJ, Brauer M, Burnett R et al. Estimates and 25-year trends of the global burden of disease attributable to ambient air pollution: an analysis of data from the Global Burden of Diseases Study 2015 [published correction appears in Lancet. 2017;389(10087):e15] [published correction appears in Lancet. 2018;391(10130):1576]. Lancet. 2017;389(10082):1907–1918. 10.1016/S0140-6736(17)30505-6. 10.1016/S0140-6736(17)30505-6 PMC5439030 28408086

[10] Zhang S, Li L, Gao W, Wang Y, Yao X. Interventions to reduce individual exposure of elderly individuals and children to haze: a review. J Thorac Dis. 2016;8(1):E62–8. 10.3978/j.issn.2072-1439.2016.01.17. 26904254 10.3978/j.issn.2072-1439.2016.01.17 PMC4740117

[11] Chen Z, Salam MT, Eckel SP, Breton CV, Gilliland FD. Chronic effects of air pollution on respiratory health in Southern California children: findings from the Southern California Children’s Health Study. J Thorac Dis. 2015;7(1):46–58. 10.3978/j.issn.2072-1439.2014.12.20. 25694817 10.3978/j.issn.2072-1439.2014.12.20 PMC4311073

[12] Patel MM, Hoepner L, Garfinkel R, et al. Ambient metals, elemental carbon, and wheeze and cough in New York City children through 24 months of age. Am J Respir Crit Care Med. 2009;180(11):1107–13. 10.1164/rccm.200901-0122OC. 19745205 10.1164/rccm.200901-0122OC PMC2784415

[13] Ryan PH, Bernstein DI, Lockey J, et al. Exposure to traffic-related particles and endotoxin during infancy is associated with wheezing at age 3 years. Am J Respir Crit Care Med. 2009;180(11):1068–75. 10.1164/rccm.200808-1307OC. 19745206 10.1164/rccm.200808-1307OC PMC2784413

[14] McConnell R, Islam T, Shankardass K, et al. Childhood incident asthma and traffic-related air pollution at home and school. Environ Health Perspect. 2010;118(7):1021–6. 10.1289/ehp.0901232. 20371422 10.1289/ehp.0901232 PMC2920902

[15] Salvi S. Health effects of ambient air pollution in children. Paediatr Respir Rev. 2007;8(4):275–80. 10.1016/j.prrv.2007.08.008. 18005894 10.1016/j.prrv.2007.08.008

[16] Lewis TC, Robins TG, Mentz GB, et al. Air pollution and respiratory symptoms among children with asthma: vulnerability by corticosteroid use and residence area. Sci Total Environ. 2013;448:48–55. 10.1016/j.scitotenv.2012.11.070. 23273373 10.1016/j.scitotenv.2012.11.070 PMC4327853

[17] Branco PT, Nunes RA, Alvim-Ferraz MC, Martins FG, Ferraz C, Vaz LG, et al. Asthma prevalence and risk factors in early childhood at Northern Portugal. Rev Port Pneumol (2006). 2016;22(3):146–50. 10.1016/j.rppnen.2015.11.001 26747645

[18] Nantanda R, Tumwine JK, Ndeezi G, Ostergaard MS. Asthma and pneumonia among children less than five years with acute respiratory symptoms in Mulago Hospital, Uganda: evidence of under-diagnosis of asthma. PLoS ONE. 2013;8(11):e81562. 10.1371/journal.pone.0081562. 24312321 10.1371/journal.pone.0081562 PMC3843700

[19] Andualem Z, Taddese AA, Azene ZN, Azanaw J, Dagne H. Respiratory symptoms and associated risk factors among under-five children in Northwest, Ethiopia: community based cross-sectional study. Multidiscip Respir Med. 2020;15(1):685. 10.4081/mrm.2020.685. 33117532 10.4081/mrm.2020.685 PMC7542992

[20] Naidoo RN, Robins TG, Batterman S, Mentz G, Jack C. Ambient pollution and respiratory outcomes among schoolchildren in Durban, South Africa. SAJCH. 2013;7(4):127–34. 25741408 10.7196/sajch.598 PMC4346135

[21] Madonsela BS, Maphanga T, Chidi BS, Shale K, Zungu V. Assessment of air pollution in the informal settlements of the Western Cape, South Africa. J Air Pollut Health. 2022;7(1):1–14.

[22] Meme H, Amukoye E, Bowyer C, Chakaya J, Das D, Dobson R, et al. Asthma symptoms, spirometry and air pollution exposure in schoolchildren in an informal settlement and an affluent area of Nairobi, Kenya. Thorax. 2023;78(11):1118–25. 37280096 10.1136/thorax-2023-220057 PMC10715514

[23] Masekela R, Vanker A. Lung Health in Children in Sub-saharan Africa: addressing the need for Cleaner Air. Int J Environ Res Public Health. 2020;17(17):6178. 10.3390/ijerph17176178. 32858786 10.3390/ijerph17176178 PMC7504680

[24] Lu W, Wang LA, Mann J, et al. Biomass smoke exposure and atopy among Young Children in the Western Highlands of Guatemala: a prospective cohort study. Int J Environ Res Public Health. 2022;19(21):14064. 10.3390/ijerph192114064. 36360942 10.3390/ijerph192114064 PMC9656762

[25] Meme H, Amukoye E, Bowyer C, et al. Asthma symptoms, spirometry and air pollution exposure in schoolchildren in an informal settlement and an affluent area of Nairobi, Kenya. Thorax. 2023;78(11):1118–25. 10.1136/thorax-2023-220057. 37280096 10.1136/thorax-2023-220057 PMC10715514

[26] Holden KA, Lee AR, Hawcutt DB, Sinha IP. The impact of poor housing and indoor air quality on respiratory health in children. Breathe (Sheff). 2023;19(2):230058. 10.1183/20734735.0058-2023. 37645022 10.1183/20734735.0058-2023 PMC10461733

[27] City of Tshwane [Internet], Stats SA. 2022 [cited 2024 May 5]. https://census.statssa.gov.za/#/province/7/2.

[28] Cooperative Governance & Traditional Affairs. City of, Tshwane. T/52.: [Internet]. City of Tshwane: [updated 2020; cited 2023 Sept 6]. https://www.cogta.gov.za/ddm/wp-content/uploads/2020/11/Tshwane-October-2020.pdf.

[29] Seneviratne R, Gunawardena NS. Prevalence and associated factors of wheezing illnesses of children aged three to five years living in under-served settlements of the Colombo Municipal Council in Sri Lanka: a cross-sectional study. BMC Public Health. 2018;18(1):127. 10.1186/s12889-018-5043-3. 29325544 10.1186/s12889-018-5043-3 PMC5765666

[30] Tai A, Volkmer R, Burton A. Prevalence of asthma symptoms and atopic disorders in Preschool Children and the Trend over a Decade. J Asthma. 2009;46(4):344–6. 10.1080/02770900802660998. 10.1080/02770900802660998 19484666

[31] Masoli M, Fabian D, Holt S, Beasley R. Global Initiative for Asthma (GINA) Program. The global burden of asthma: executive summary of the GINA Dissemination Committee report. Allergy. 2004;59(5):469–78. 10.1111/j.1398-9995.2004.00526.x. 15080825 10.1111/j.1398-9995.2004.00526.x

[32] Sheuya SA. Improving the health and lives of people living in slums. Ann N Y Acad Sci. 2008;1136:298–306. 10.1196/annals.1425.003. 17954669 10.1196/annals.1425.003

[33] Webber MP, Carpiniello KE, Oruwariye T, Appel DK. Prevalence of asthma and asthma-like symptoms in inner-city elementary schoolchildren. Pediatr Pulmonol. 2002;34(2):105–11. 10.1002/ppul.10146. 12112776 10.1002/ppul.10146

[34] Demoulin-Alexikova S, Plevkova J, Mazurova L, Zatko T, Alexik M, Hanacek J, Tatar M. Impact of Air Pollution on age and gender related increase in Cough Reflex sensitivity of healthy children in Slovakia. Front Physiol. 2016;7:54. 10.3389/fphys.2016.00054. 26941651 10.3389/fphys.2016.00054 PMC4763033

[35] Siddique S, Ray MR, Lahiri T. Effects of air pollution on the respiratory health of children: a study in the capital city of India. Air Qual Atmos Health. 2011;4(2):95–102. 10.1007/s11869-010-0079-2. 32215114 10.1007/s11869-010-0079-2 PMC7089414

[36] Shirinde J, Wichmann J, Voyi K. Allergic rhinitis, rhinoconjunctivitis and hayfever symptoms among children are associated with frequency of truck traffic near residences: a cross sectional study. Environ Health. 2015;14:84. 10.1186/s12940-015-0072-1. 26503217 10.1186/s12940-015-0072-1 PMC4620607

[37] Giovanis E. Relationship between recycling rate and air pollution: Waste management in the state of Massachusetts. Waste Manag. 2015;40:192–203. 10.1016/j.wasman.2015.03.006. 25827258 10.1016/j.wasman.2015.03.006

[38] Sennès V, Gombert-Courvoisier S, Félonneau ML, Ribeyre F. Citizens’ environmental awareness and responsibility at local level. Int J Urban Sustain Dev. 2012;4(2):186–97. 10.1080/19463138.2012.694819. 10.1080/19463138.2012.694819

[39] Xu J, Chi CSF, Zhu K. Concern or apathy: the attitude of the public toward urban air pollution. J Risk Res. 2017;20:482–98. 10.1080/13669877.2015.1071869. 10.1080/13669877.2015.1071869

[40] Ramírez AS, Ramondt S, Van Bogart K, Perez-Zuniga R. Public Awareness of Air Pollution and Health threats: challenges and opportunities for Communication Strategies to Improve Environmental Health Literacy. J Health Commun. 2019;24(1):75–83. 10.1080/10810730.2019.1574320. 30730281 10.1080/10810730.2019.1574320 PMC6688599

[41] Ghorani-Azam A, Riahi-Zanjani B, Balali-Mood M. Effects of air pollution on human health and practical measures for prevention in Iran. J Res Med Sci. 2016;21:65. 10.4103/1735-1995.189646. 27904610 10.4103/1735-1995.189646 PMC5122104

[42] Kelly FJ, Fussell JC. Air pollution and public health: emerging hazards and improved understanding of risk. Environ Geochem Health. 2015;37(4):631–49. 10.1007/s10653-015-9720-1. 26040976 10.1007/s10653-015-9720-1 PMC4516868

[43] Taniguchi Y, Yamazaki S, Michikawa T, Nakayama SF, Sekiyama M, Nitta H, Mezawa H, Saito-Abe M, Oda M, Mitsubuchi H, Sanefuji M, Ohga S, Mise N, Ikegami A, Shimono M, Suga R. Associations of dog and cat ownership with wheezing and asthma in children: pilot study of the Japan Environment and children’s study. PLoS ONE. 2020;15(5):e0232604. 10.1371/journal.pone.0232604. 32407337 10.1371/journal.pone.0232604 PMC7224482

[44] Rives R, Elshorbany Y, Kaylor S. The relationship between Air Quality, Health outcomes, and socioeconomic impacts of the COVID-19 pandemic in the US. Geohealth. 2023;7(5):e2022GH000735. 10.1029/2022GH000735. 37181011 10.1029/2022GH000735 PMC10171069

