# Supplementary material for: Respiratory conditions and health symptoms associated with air pollution amongst children aged six years and below in Melusi Informal Settlement, Tshwane Metropolitan Municipality, South Africa: a cross-sectional study
Source: BMC Public Health. 2024 Jul 30;24:2038. doi: 10.1186/s12889-024-19324-w (PMC11290171; doi:10.1186/s12889-024-19324-w)
Supplement: Supplementary file 1 — Supplementary Material 1 [file 12889_2024_19324_MOESM1_ESM.docx]

# ISAAC
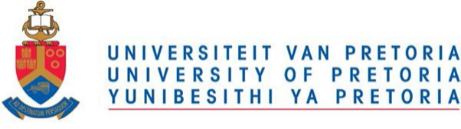


University of Pretoria

# Questionnaire

**Instructions for completing the questionnaire**

Examples of instructions for completing questionnaire and demographic questions are given below.

On this sheet are questions about your child’s name, school, and birth dates. Please write your answers to these questions in the space provided.

All other questions require you to tick your answer in a box. If you make a mistake, put a cross in the box and tick the correct answer. Tick only one option unless otherwise instructed.

4

Examples of how to mark questionnaires: Age years

To answer Yes/No, put a cross in the appropriate box as per example below:

| YES |  |
| --- | --- |
| NO | **X** |

----------------------------------------------------------------------------------------------------------------------------------------------------------

**SUBURB/ TOWNSHIP/SECTION**

**WHERE YOUR CHILD LIVES**:

**YOUR CHILD’S SCHOOL**:

**TODAY’S DATE**:

**YOUR CHILD’S NAME**: (code sheet will be used to

Record the names separately – names will not

for be used reporting )

**YOUR CHILD’S AGE**: years

**YOUR CHILD’S DATE OF BIRTH**:

**WEIGHT OF YOUR CHILD**? kg

**HEIGHT OF YOUR CHILD?**  Metres /centimeters (Please circle the measurement you used)

**(Tick all your answers for the rest of the questionnaire)**

**Is your child a**: **MALE**  **FEMALE**

**Core questionnaire for asthma**

1. Has your child ever had wheezing or whistling in the chest at any time in the past?

| YES |  |
| --- | --- |
| NO |  |

## IF YOU HAVE ANSWERED “NO” PLEASE SKIP TO QUESTION 6

1. Has your child had wheezing or whistling in the chest in the past 12 months?

| YES |  |
| --- | --- |
| NO |  |

## IF YOU HAVE ANSWERED “NO” PLEASE SKIP TO QUESTION 6

1. How many attacks of wheezing has your child had in the past 12 months?

| NONE |  |
| --- | --- |
| 1 TO 3 |  |
| 4-14 |  |
| More than 12 |  |

1. In the past 12 months, how often, on average, has your child’s sleep been disturbed due to wheezing?

| Never woken with wheezing |  |
| --- | --- |
| Less than one night per week |  |
| One or more nights per week |  |

1. In the past 12 months, has wheezing ever been severe enough to limit your child’s speech to only one or two words at a time between breaths?

| YES |  |
| --- | --- |
| NO |  |

1. Has your child ever had asthma?



| YES |  |
| --- | --- |
| NO |  |

1. Was the asthma diagnosed by a medical doctor or nurse?

| YES |  |
| --- | --- |
| NO |  |

1. In the past 12 months, has your child’s chest ever sounded wheezy during or after playing?

| YES |  |
| --- | --- |
| NO |  |

1. In the past 12 months, has your child had a dry cough at night, apart from a cough associated with a cold or chest infection?

| YES |  |
| --- | --- |
| NO |  |

**Core questionnaire for rhinitis**

1. Has your child ever had a problem with sneezing, or a runny, or blocked nose when she/he DID NOT have a cold or the flu?

| YES |  |
| --- | --- |
| NO |  |

## IF YOU HAVE ANSWERED “NO” PLEASE SKIP TO QUESTION 14

1. In the past 12 months, has your child had a problem with sneezing, or a runny, or blocked nose when she/he DID NOT have a cold or the flu?

| YES |  |
| --- | --- |
| NO |  |

## IF YOU HAVE ANSWERED “NO” PLEASE SKIP TO QUESTION 14

1. In the past 12 months, has this nose problem been accompanied by itchy-watery eyes?

| YES |  |
| --- | --- |
| NO |  |

1. In which of the past 12 months did this nose problem occur? (Please tick any which apply)

| January |  |
| --- | --- |
| February |  |
| March |  |
| April |  |
| May |  |
| June |  |
| July |  |
| August |  |
| September |  |
| October |  |
| November |  |
| December |  |

1. In the past 12 months, how much did this nose problem interfere with your child’s daily activities?

| Not at all |  |
| --- | --- |
| A little |  |
| A moderate amount |  |
| A lot |  |

1. Has your child ever had hayfever?



| YES |  |
| --- | --- |
| NO |  |

1. Was the hayfever diagnosed by a doctor or nurse?

| YES |  |
| --- | --- |
| NO |  |

**Core questionnaire for eczema**

1. Has your child ever had an itchy rash, which was coming and going for at least six months?

| YES |  |
| --- | --- |
| NO |  |

## IF YOU HAVE ANSWERED “NO” PLEASE SKIP TO QUESTION 21

1. Has your child had this itchy rash at any time in the past 12 months?

| YES |  |
| --- | --- |
| NO |  |

## IF YOU HAVE ANSWERED “NO” PLEASE SKIP TO QUESTION 21

1. Has this itchy rash at any time affected your child on any of the following places:

the folds of the elbows, behind the knees, in front of the ankles, under the buttocks,

or around the neck, ears or eyes?

| YES |  |
| --- | --- |
| NO |  |

1. At what age of the child did this itchy rash first occur?

| Age 2-4 years |  |
| --- | --- |
| Age 5 or more |  |
| Can’t remember |  |

1. Has this rash cleared completely at any time during the past 12 months?

| YES |  |
| --- | --- |
| NO |  |

1. In the past 12 months, how often, on average, has your child been kept awake at night by this itchy rash?

| Never in the past 12 months |  |
| --- | --- |
| Less than one night per week |  |
| One or more nights per week |  |

1. Has your child ever had eczema?



| YES |  |
| --- | --- |
| NO |  |

1. Was the eczema diagnosed by a doctor or nurse?

| YES |  |
| --- | --- |
| NO |  |

**General questionnaire**

1. How long has your child lived in this / TOWNSHIP/SECTION?

| Less than 6 months |  |
| --- | --- |
| 6 to 12 months |  |
| 1 to 2 years |  |
| 3 years or longer |  |

1. How does your child usually get to school?

| Walk |  |
| --- | --- |
| Taxi/Bus |  |
| Motor car |  |
| Combination |  |
| Other |  |

1. How far is the nearest Clinic or Hospital from your home?

| 15 minutes’ walk or 5 minute drive |  |
| --- | --- |
| 1 hour walk or 15 minute drive |  |
| more than an hour’s walk or more than  30 minute drive |  |

1. What type of house does your child live in?

| Brick |  |
| --- | --- |
| Mud |  |
| Corrugated iron |  |
| Combination |  |
| Other |  |

1. How many rooms are in your house? (Excluding bathrooms)

|  |
| --- |

1. Do you have running water in the house?

| YES |  |
| --- | --- |
| NO |  |

1. In the past 12 months, how often, on average, did your child eat or drink the following?: (Please leave blank if you do not know what a food is)

| **Type of food** | **Never or occasionally** | **Once or twice per week** | **Three or more times per week** |
| --- | --- | --- | --- |
| Meat (e.g. beef, lamb, chicken, pork) |  |  |  |
| Seafood (including fish) |  |  |  |
| Fruit |  |  |  |
| Vegetables (green and root) |  |  |  |
| Pulses (peas, beans, lentils) |  |  |  |
| Cereal (including bread) |  |  |  |
| Pasta |  |  |  |
| Rice |  |  |  |
| Butter |  |  |  |
| Margarine |  |  |  |
| Nuts |  |  |  |
| Potatoes |  |  |  |
| Milk |  |  |  |
| Eggs |  |  |  |
| Fast food/burgers |  |  |  |

1. In the past 6 months, how often has your child been absent from school?

| Never or occasionally |  |
| --- | --- |
| Once or twice per week |  |
| Three or more times a week |  |

1. During a normal week, how many hours a day (24hours) does your child watch television?

| Less than 1 hour |  |
| --- | --- |
| 1 hour but less than 3 hours |  |
| 3 hours but less than 5 hours |  |
| 5 hours or more |  |

1. In your house, what fuel is usually used for cooking?

| Electricity |  |
| --- | --- |
| Gas |  |
| Paraffin |  |
| Open fires |  |
| Other – Please specify |  |

1. In your house, what fuel is usually used for heating?

| Electricity |  |
| --- | --- |
| Gas |  |
| Paraffin |  |
| Open fires (wood, coal) |  |
| Other – Please specify |  |

1. In the past 12 months, how often, on average, have you given your child paracetamol (e.g. Panado , Pamol)?

| Never |  |
| --- | --- |
| At least once a year |  |
| At least once per month |  |

13. How many older brothers and sisters does your child have?

| Brothers |  |
| --- | --- |
| Sisters |  |

14. How many younger brothers and sisters does your child have?

| Brothers |  |
| --- | --- |
| Sisters |  |

15. Was your child born in this township/suburb?

| YES |  |
| --- | --- |
| NO |  |

16. How often do trucks pass through the street where you live, on weekdays?

| Never |  |
| --- | --- |
| Seldom |  |
| Frequently through the day |  |
| Almost all day |  |

17. Do you currently have a cat in your home?

| YES |  |
| --- | --- |
| NO |  |

18. In the past 12 months, have you had a cat in your home?

| YES |  |
| --- | --- |
| NO |  |

19. Do you currently have a dog in your home?

| YES |  |
| --- | --- |
| NO |  |

20. In the past 12 months, have you had a dog in your home?

| YES |  |
| --- | --- |
| NO |  |

21. Does the child’s mother (or female guardian) smoke cigarettes?

| YES |  |
| --- | --- |
| NO |  |

22. Does the child’s father (or male guardian) smoke cigarettes?

| YES |  |
| --- | --- |
| NO |  |

23. How many people living in the same house as your child smoke cigarettes?

|  |
| --- |

People

24. In the past 30 days about how many days would you say your child was in a place where someone smoked close to him/her (no complete physical barrier i.e. smoke got to him/her)?

|  | Never | 1-6 days | 7-10 days | 16-20 days | More than 20 days |
| --- | --- | --- | --- | --- | --- |
| At home |  |  |  |  |  |
| At school |  |  |  |  |  |
| In the car or transport |  |  |  |  |  |
| Restaurant |  |  |  |  |  |

**Air quality perceptions**

25. How would you rate the indoor air quality in your home?

| Good |  |
| --- | --- |
| Average |  |
| Poor |  |

1. How would you rate the outdoor air quality in your community?

| Good |  |
| --- | --- |
| Average |  |
| Poor |  |

26. **Please express how much do you agree or disagree on the following statements**

|  | **Strongly agree** | **Agree** | **Undecided** | **Disagree** | **Strongly disagree** |
| --- | --- | --- | --- | --- | --- |
| Improving environment is the responsibility of every citizen |  |  |  |  |  |
| Recycling programs should be put in place and promoted across the whole city |  |  |  |  |  |
| I am actively involved in cleaning up the environment |  |  |  |  |  |
| The pollution is out of my control and I cannot do anything to change it |  |  |  |  |  |
| If I knew how to better contribute to a cleaner environment, I would take action |  |  |  |  |  |
| I do not see the pollution as a health problem |  |  |  |  |  |

**THANK YOU!**
